# Should we be Prescribing Fluphenazine Long-Acting Injectable Formulation?

**DOI:** 10.1007/s11920-025-01610-y

**Published:** 2025-04-28

**Authors:** Dustin Rowland

**Affiliations:** Department of Psychiatry, University of Michigan, 4250 Plymouth Rd, Ann Arbor, MI 48105 US

**Keywords:** Fluphenazine, Long-acting injectable, First-generation antipsychotic, Social determinants of health (SDoH), Antipsychotic tolerability, Treatment cost efficacy

## Abstract

**Purpose of Review:**

This review critically examines the clinical utility, efficacy, and tolerability of fluphenazine long-acting injectable (LAI) relative to contemporary alternatives. It further evaluates whether fluphenazine LAI confers substantive advantages over other available formulations for the management of schizophrenia, particularly in light of its long-standing use.

**Recent Findings:**

The extant literature demonstrates that the tolerability and side effect profile of fluphenazine LAI are comparable to other FGA LAIs but likely less favorable than available second-generation antipsychotic (SGA) LAIs. Although fluphenazine trends towards the higher end of the efficacy scale in meta-analyses, there is a lack of robust evidence showing a true statistical superiority for relapse prevention in schizophrenia. Social determinants of health (SDoH), such as race and economic factors, significantly influence its prescribing patterns.

**Summary:**

Fluphenazine LAI continues to be utilized primarily due to its low cost and widespread clinical familiarity rather than evidence-based superiority in efficacy or tolerability. Its prescribing is disproportionately influenced by healthcare inequities and resource limitations. Clinicians should employ a rigorous, individualized approach to antipsychotic selection, incorporating shared decision-making and patient education to ensure optimal therapeutic outcomes.

## Introduction

Fluphenazine is a phenothiazine high-potency typical antipsychotic that is theorized – like most antipsychotics – to primarily assert both its therapeutic effects and side effects via alteration of D2 receptor activity within the mesolimbic and nigrostriatal pathways [1]. This antipsychotic has a comparatively long history of use in the United States, being the third antipsychotic approved by the FDA. Its 1959 oral formulation approval was preceded only by the approvals of oral perphenazine in 1957 and oral chlorpromazine in 1954 [2]. Nine years after the oral formulation of fluphenazine was approved, the long-acting injectable (LAI) “depot” formulation was approved – the first approval of an antipsychotic LAI by the FDA [3]. Until the approval of depot formulation haloperidol in 1986, this was the only long-acting injectable available on the market [4]. And, until 2021, fluphenazine was the only long-acting injectable on the WHO essential medicine list [5]. Early studies indicated that this long-acting formulation was at least as effective as oral antipsychotics for management of schizophrenia, although contemporary articles recommended caution with dosing amounts and intervals to minimize side effects – as well as taking individual patient history into account [6].

Although fluphenazine LAI has a long history of being used for treatment of schizophrenia, its use has declined as other, ostensibly more benign or efficacious, depot formulations have entered the market [7]. However, there have been very few studies specifically evaluating fluphenazine LAI head-to-head against newer options [8]. A common theme within the literature is the postulate that the primary driving factor behind the continued use of fluphenazine LAI – both within the United States and worldwide - is its lower cost [9, 10]. This concept is rarely supported in a robust way, which may be related to the notable lack of high-quality studies on fluphenazine LAI, despite its nearly six decades of clinical utilization [1]. The purpose of this article is to collate and discuss the extant data on fluphenazine LAI, with an aim to provide practical clinical information.

### Cost

**Fig. 1:**
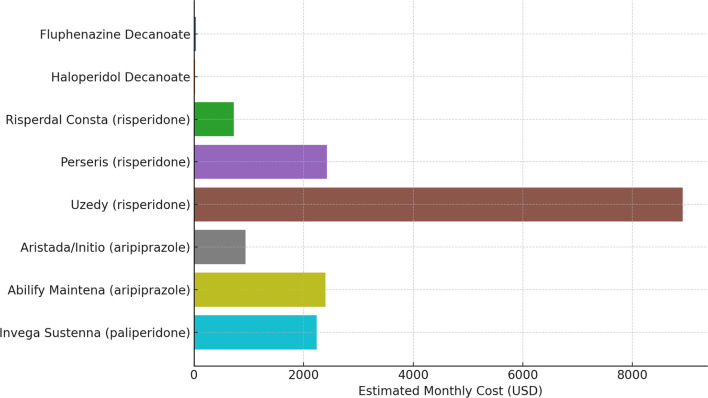
Average price per month of long acting injectable antipsychotic medications (NADAC, 2025)

A search of the Medicaid National Average Drug Acquisition Cost database for 2025 showed significant disparities in price of long-acting injectable medications. Fluphenazine decanoate remains the cheapest formulation, at an average price of $16. Haloperidol is very similar at $23 – and it bears noting here that generally fluphenazine decanoate is given at 2 week intervals, and therefore the monthly cost of haloperidol decanoate is actually lower, even without applying system costs for injection administration. Of the atypical LAIs available, risperidone ER microspheres (generic for Risperdal Consta) was the cheapest at $489-$968 depending on dose. Newer subcutaneous risperidone formulations Uzedy and Perseris were priced at $8772–9061 and $2077–2767, respectively. Risvan and Rykindo risperidone LAIs were not listed in the NADAC, likely due to recent FDA approval. Aripiprazole depot formulations ranged from $907–967 for Aristada ER and Aristada Initio to $2019–2778 for Abilify Maintena; all doses of paliperidone LAI were $2235 [11]. Paliperidone three- and six-month versions have also been approved. For clarity, this paper will focus on the options that have more clinical data and a lower price point. A visual comparison of these options is given below in Fig. 1.

As noted above, not all long-acting injectables have the same dosing interval – although clinically, the intervals are often adjusted to target refractory symptoms. The half life of fluphenazine decanoate is roughly 1 week, and it is most frequently given as an injection every 2 weeks. There are reports that fluphenazine reaches peak serum levels rapidly, although the exact rate varies considerably in the literature. One experimental paper from 1979 noted peak serum levels were reached between 1 and 8 h after injection, with significant variation between patients [12]. Another experimental paper from 1985 noted peak serum levels occurring generally in less than 24 h, again with a very significant variation between patients [13]. This phenomenon has been noted essentially since the approval of the LAI [14]. The specific applications of this phenomenon vary between clinicians, with some reporting no need for oral overlap - especially in cases of likely patient non-adherence - while others recommend an oral overlap during the first two weeks after initiation [9, 15]. There have been no well-controlled studies of either technique, although a retrospective data analysis noted heterogeneity in clinician application of oral overlap [16]. The biweekly dose of fluphenazine decanoate given is 1.25 times the daily oral dose. A q4week depot version of fluphenazine exists, but the risks of this enanthate formulation included high risk of depression, EPS, and possibly hypotension and other serious cardiac side effects [17, 18]. To briefly compare to other LAI options discussed above, only the risperidone formulation traditionally requires a q2week injection frequency; haloperidol is also dissolved in oil and therefore these first-generation LAIs are more painful to inject [9].

### LAI Trends Over Time

Although there is no publicly accessible way to globally track prescribing trends for various formulations of antipsychotic injectables, multiple articles have noted an overall trend away from first-generation antipsychotic (FGA) LAI prescriptions. The putative cause for this transition is the improved side effect profiles of newer agents, which are presumed to lead to better adherence to treatment plans [19, 20]. One 2017 study by Haddad & Olajide specifically analyzed extant data for the interval between 2002 and 2008. During this period, there was an overall decrease in prescription of fluphenazine LAI, within the context of a general trend away from FGA LAIs due to the increasing availability and usage of risperidone LAI formulations [21]. A 2023 article by Yasui-Furukori et al. reviewed data on a single patient cohort between 2001 and 2021. In this analysis, there was a clear trend demonstrating reduction in FGA LAIs with a commensurate increase in usage of second-generation antipsychotic (SGA) LAIs. However, the authors noted that patients who had been initially stabilized on a first-generation antipsychotic were less likely to be transitioned to a second-generation antipsychotic [22].

### Side Effects

As noted above, concern about side effects and resulting non-adherence was considered a driver of the transition from FGAs to SGAs in LAI prescribing trends. The data specifically comparing fluphenazine to other antipsychotics in terms of side effects, especially the LAI formulation, is surprisingly thin given the long history of fluphenazine’s use for treatment of schizophrenia. However, articles that discuss sedation, mood, anticholinergic effects, corrected QT interval (QTc) prolongation and other cardiac effects, EPS, weight gain, and overall tolerability of fluphenazine were identified. Unfortunately, data obtained from trials of PO medication is difficult to generalize to LAI formulations of the same medication; for example, a large 2021 analysis of antipsychotics by Eugene et al. showed that haloperidol LAI was significantly less sedating than an equivalent dose of oral haloperidol. The same analysis rated oral fluphenazine as among the most sedating antipsychotics, but excluded fluphenazine LAI due to lack of comparative data [23].

In terms of mood, there are some older heuristic indications that fluphenazine was believed to have a lower risk of causing depression than other antipsychotics. However, a 1986 trial of haloperidol and fluphenazine indicated haloperidol was overall superior in terms of depression risk [24]. Anticholinergic effects are another consideration when choosing fluphenazine against other LAI options, but a large 2015 meta-analysis by Maayan et al. did not identify a significant difference in anticholinergic effects between LAIs, including fluphenazine [25]. Data related to QTc prolongation is especially thin; a 2000 Lancet article reported that fluphenazine did not cause appreciable QTc prolongation. However, this conclusion appears to be based on the 95% confidence interval containing a no-effect value; further evaluation of the data shows that there is no significant difference shown between fluphenazine, haloperidol, risperidone, and chlorpromazine. As these other agents are generally considered QTc-prolonging, it is difficult to imagine that fluphenazine truly does not cause QTc prolongation [26]. However, within the Mayaan et al. meta-analysis, there was no correlation between fluphenazine use and increased risk of lethal events [25]. This may be due to poor correlation between average QTc prolongation and risk of developing Torsades de Pointes [27].

Since early after its approval, fluphenazine has been noted to have a high incidence of extrapyramidal symptoms (EPS) in both oral and depot forms. A 1976 study found that 65% of patients on fluphenazine required benztropine for management of extrapyramidal side effects. This study, which included 93 patients on oral fluphenazine and 102 patients on depot fluphenazine, did not find a difference in EPS between oral and LAI formulations [14]. In fact, multiple recent meta-analyses have noted that fluphenazine is one of the antipsychotics most implicated in both akathisia and use of anti-parkinsonian medication [28, 29]. However, although fluphenazine LAI and haloperidol LAI are both more likely to cause EPS than most other agents, another meta-analysis noted that the rates of EPS were comparable to those with risperidone LAI.

Although multiple older articles cite reduced risk of weight gain as a reason to choose fluphenazine LAI over a second-generation antipsychotic, there does not appear to be enough available data to support this conclusion. The Lancet meta-analysis excluded fluphenazine from its weight gain analysis due to lack of data [29]. A single 1986 study did compare haloperidol LAI and fluphenazine LAI head to head across numerous categories, including weight gain. That study did not find a statistically significant difference between either of these agents although it noted a trend towards superiority for haloperidol [30].

In terms of prolactin elevation, although there are some small studies that indicate fluphenazine may be more prolactin-increasing than atypical antipsychotics there is an unfortunate lack of robust comparative data for fluphenazine that would allow for a meta-analysis [31, 32]. 

The key reason for detailed analysis of antipsychotic side effects is predicting overall tolerability within patient populations, as adherence issues are generally deemed to stem from poor tolerance. Improved adherence eclipses most other factors in terms of overall long-term outcomes in schizophrenia treatment [21]. Despite the concerns with EPS and other side effects generally associated with poor tolerability, there was not a statistically significant difference in drop out rates for patients on fluphenazine LAI vs. other LAI options. This correlation held true both in the short term - despite higher rates of reported adverse effects with fluphenazine LAI - and in the long term, with the inclusion of studies greater than 1 year in duration [25]. However, a separate meta-analysis that included risperidone LAI noted that although most antipsychotics, including fluphenazine LAI, are less tolerable than placebo, the data did not show a drop out rate for risperidone LAI greater than in the placebo group [33].

Overall, there does not appear to be any robust evidence that fluphenazine has superiority in terms of side effects in any specific area, including historically considered categories such as mood symptoms and weight gain. The data on EPS and overall tolerability is mixed, but certainly does not favor fluphenazine as superior to other available LAI options.

### Efficacy

Although side effects can be very influential in modifying patients’ adherence to their regimen, which is a very key marker associated with improved outcomes, efficacy is itself modified by numerous other factors. A analysis in *The Lancet* published by Schneider-Thoma et al. compared 32 antipsychotic formulations, including nine LAIs, in terms of relative risk of relapse (RRR). The 95% credible interval (CrI) for fluphenazine LAI RRR was 0.22 (0.12 to 0.35), ranking it as the 5th overall antipsychotic in terms of efficacy for relapse prevention. Interestingly, the only LAI to rank higher on this scale was the zuclopenthixol LAI, which is not FDA approved for use in the United States, with a RRR of 0.07 (0.00 to 0.34). However, every 95% credible interval for all 31 other antipsychotics, oral and LAI, was overlapping with both the fluphenazine LAI and the zuclopenthixol LAI, with the very lowest ranking treatment option - cariprazine oral - having a 95% CrI of 0.65 (0.16 to 1.14) [29]. A separate systematic review by Mayaan et al. comparing fluphenazine LAI to other available LAIs noted no evidence for a statistically significant different in patient relapse events [25]. One additional systematic review and meta-analysis by Zhao et al. found that the only antipsychotic treatment significantly worse than fluphenazine LAI in terms of relapse rate was oral chlorpromazine, which within the meta analysis was the lowest performing antipsychotic [33]. Groenendaal et al. notes that generally, studies comparing FGA and SGA LAI formulations do not show statistically significant differences in relevant outcomes [2]. Unfortunately, this is a common problem within psychiatric research, as the large number of contributing external factors - that are difficult to quantify and adjust for - makes statistical significance an exceedingly challenging target to reach. However, within the large data sets, reviews, and analyses available, there certainly is not sufficient data to support fluphenazine LAI as an objectively superior agent for relapse prevention.

### Social Determinants of Health

If the data for fluphenazine does not support its superiority in terms of any specific side effect, tolerability, or overall prevention of relapse, then what drives the continuing use of this antipsychotic? As per Fig. 1 above, there exists an order of magnitude difference in price between first and second generation antipsychotic depot formulations. Without specifically interpreting the data, it bears notice that there is a strong positive correlation - worldwide - between mean income and use of second generation antipsychotics [34]. In addition, patients with stigmatized conditions or situations, such as those who have substance use disorders or are undomiciled, are much more likely to receive first-generation LAIs. This correlation is especially strong for fluphenazine LAI, with 60% of patients receiving this formulation being unhoused. Within the undomiciled population, any use of second-generation LAI, such as aripiprazole-based formulations, was heavily skewed towards white patients [2]. A 2003 analysis by Kreyenbuhl et al. noted a similar very strong correlation between race of a patient with schizophrenia and which generation of antipsychotic injectable that patient received. This analysis was careful to note that correction for well-known confounding factors such as age, gender, and local formulary did not mitigate the correlation, with black patients still receiving first-generation LAIs up to 600% more often than white patients [35].

### SDoH and Antipsychotic Selection

It is likely that fluphenazine LAI is being started on inpatient units due to its very low cost and lack of need for an oral overlap, with heavier use within psychiatric hospitals who see a large number of patients and are under pressure to provide rapid discharges. Given the lack of any clear non-cost advantage to using fluphenazine LAI long-term, it may be that clinicians are unnecessarily continuing this medication due to inertia, as well as concerns about resurgent psychosis [33]. It may be that, especially in community mental health settings where resources are tight, use of cheaper LAIs is reasoned to be a way to preserve capital for other sectors, such as case management. Additionally, response to any medication in schizophrenia is driven to some extent by patient-specific factors; despite this, there is no clear rationale for using fluphenazine prior to trials of numerous other agents.

### Changing LAIs - Risks and Benefits

Clinicians receiving patients on fluphenazine LAI should be conscious of the data as outlined above, as well as the risks and benefits of switching antipsychotics. Patient education about the available options and shared decision-making will be key to determining whether a change of antipsychotic formulation aligns with the patient’s values. Note that even if second-generation antipsychotic medications are not an option due to resource constraints, there is not a clear cost superiority of fluphenazine LAI over haloperidol LAI. In addition, haloperidol LAI can be given at longer intervals, reducing system costs and staff burden [36]. The risk of destabilization when switching medications in a stable patient should be considered as well as discussed with the patient; some patients may prefer to stay with the medication they are familiar with. One study specifically noted that patients whose psychosis had been stabilized with fluphenazine LAI were less likely to be switched to a second-generation antipsychotic than other similar patients [22]. A last consideration is the lack of robust, tested protocols for switching between long-acting injectable formulations; the switching strategy should be guided by balancing the severity of the patient’s psychotic episodes with the general side effect burden.

## Summary and Conclusion

Fluphenazine is one of the oldest oral antipsychotics and has the oldest FDA-approved depot formulation. Low cost, rapidly reached peak plasma concentrations, and clinicians with extensive experience utilizing fluphenazine LAI drive continued use of this formulation, although overall use of first-generation antipsychotics continues to decrease. Old heuristic notions of fluphenazine as less likely to cause weight gain, sedation, or depression are not supported by the available evidence. Additionally, the most commonly use fluphenazine LAI dosing protocol requires administration every two weeks, increasing overall system burden. There is unfortunately a dearth of robust evidence for comparison of fluphenazine LAI to newer agents, but the modest evidence that does exist tends to favor other agents in terms of acceptability. Despite this, SDoH are clearly implicated in prescribing patterns for long-acting injectable antipsychotics, with fluphenazine use being skewed towards poorer communities of color, undomiciled patients, and patients with substance use disorders. Due to this, a clinician receiving a patient who historically has been administered fluphenazine LAI should consider this a “red flag” to carefully scrutinize the patient’s medication regimen. Shared decision-making with the patient as well as psychoeducation on options, risks, and benefits is key. This should especially take individual patient factors, including tolerance to the medication, into account to support adherence as well as long-term outcomes.

### Key References

- Groenendaal E, Lynch S, Dornbush R, Klepacz L, Ferrando S. Clinical determinants, patterns and outcomes of antipsychotic medication prescribing in the treatment of schizophrenia and schizoaffective disorder: a naturalistic cohort study. J Psychiatr Res. 2023;158:273–80.
-
  - This study is important due to the recency of its publishing and the utility of the included data for linking important clinical outcomes to social determinants of health
- Maayan N, Quraishi SN, David A, Jayaswal A, Eisenbruch M, Rathbone J, et al. Fluphenazine decanoate (depot) and enanthate for schizophrenia. Cochrane Database Syst Rev. 2015;(2).
-
  - This reference provides a very detailed analysis of the extant data on fluphenazine LAI options at a date after the widespread availability of other commonly used doptions.
- Schneider-Thoma J, Chalkou K, Dörries C, Bighelli I, Ceraso A, Huhn M, et al. Comparative efficacy and tolerability of 32 oral and long-acting injectable antipsychotics for the maintenance treatment of adults with schizophrenia: a systematic review and network meta-analysis. Lancet. 2022;399(10327):824–36.
-
  - This reference is of key importance due to the recency of its publishing, the rigor of its analyses, and the large number of included medications.
- Zhao YJ, Lin L, Teng M, Khoo AL, Soh LB, Furukawa TA, et al. Long-term antipsychotic treatment in schizophrenia: systematic review and network meta-analysis of randomised controlled trials. BJPsych Open. 2016;2(1):59–66.
-
  - This reference, although not as recent as another meta-analysis cited, is an earlier robust comparison of extant RCTs at its time of publication.

## Data Availability

No datasets were generated or analysed during the current study.
